# Evaluation of the cytotoxicity and phototoxicity of *Caryocar brasiliense* supercritical carbon dioxide extract

**DOI:** 10.1186/1472-6882-14-450

**Published:** 2014-11-18

**Authors:** Lilian FB Amaral, Patricia Moriel, Mary Ann Foglio, Priscila G Mazzola

**Affiliations:** Department of Clinical Pathology, Faculty of Medical Sciences, University of Campinas (FCM-UNICAMP), Rua Tessália Vieira de Camargo, 126 Campinas, SP Brasil; CPQBA - Multidisciplinary Center of Chemical, Biological and Agricultural Researches, University of Campinas (UNICAMP), Rua Alexandre Cazellato, 999, Vila Betel, Paulínia, SP Brasil

**Keywords:** Pequi, Supercritical CO_2_ extraction, Cytotoxicity, Phototoxicity

## Abstract

**Background:**

*Caryocar brasiliense* Camb (Pequi) is a typical Brazilian Cerrado fruit tree. Its fruit is used as a vitamin source for culinary purposes and as a source of oil for the manufacture of cosmetics. *C. brasiliense* supercritical CO_2_ extracts exhibit antimicrobial activity against the bacteria *Escherichia coli*, *Pseudomonas aeruginosa*, and *Staphylococcus aureus* and also possess antioxidant activity. This study was designed to evaluate the *in vitro* cytotoxicity and phototoxicity of the supercritical CO_2_ extract obtained from the leaves of this species.

**Methods:**

*In vitro* cytotoxicity and phototoxicity of *C. brasiliense* supercritical CO_2_ extracts were assessed using a tetrazolium-based colorimetric assay (XTT) and Neutral Red methods.

**Results:**

We found that the *C. brasiliense* (Pequi) extract obtained by supercritical CO_2_ extraction did not present cytotoxic and phototoxic hazards.

**Conclusions:**

This finding suggests that the extract may be useful for the development of cosmetic and/or pharmaceutical products.

## Background

Medicinal plants have been the subject of intense research due to their potential use in cosmetic and pharmaceutical actives and also due to the growing consumer interest in using less "aggressive" products derived from natural sources. There is considerable published data regarding the efficacy of plant extracts, but information on the toxicity of these natural resources is not sufficient [1]. A careful screening of plant extracts for their activities against microorganisms or potential diseases is required to ascertain their toxic potential [2].

During the last decade, *in vitro* studies of cell and tissue cultures have become important approaches to understand the consequences of exposure and to assign risk. *In vitro* tests are commonly used for product development, drug discovery, and safety evaluation [3].

*In vitro* cytotoxicity and phototoxicity methods have been evaluated as means to reduce and refine the use of animals in testing procedures. These methods may be helpful for predicting acute toxicity *in vivo* [4].

Cytotoxicity has been defined as the adverse effects resulting from interference with the structures and/or processes essential for the survival, proliferation, and/or function of cells [5]. Grisham and Smith [6] also concluded that *in vitro* cytotoxicity assays may be useful for the prediction of acute lethal potency since the actions of substances that produce injury and death are exerted ultimately at the cellular level.

Phototoxicity is an acute reaction that can be induced by single treatment with a chemical and ultraviolet (UV) or visible radiation. Photoirritation is used to describe phototoxic reactions in the skin due to topically-applied substances combined with light exposure [7].

*Caryocar brasiliense* Camb (Pequi) is a typical Brazilian Cerrado fruit tree [8]. Its fruit is used as a vitamin source for culinary purposes and as a source of oil for the manufacture of cosmetics [9]. "Pequi" (originates from the Tupi–Guarani language) means "spiny-skinned fruit", which refers to a shell covered with thin woody spikes, protecting the seeds [10].

Pequi oil is employed for the treatment of hoarseness, sore throat, bronchitis, and cough. It is used topically for dressing wounds as well as for relieving muscle aches, rheumatic pains, and contusions [11]. It is also used for lung infections and has veterinary indications [12]. It can be employed against respiratory problems and scarring [13]. Pequi oil has anti-inflammatory activity [14] and can be used as an aphrodisiac as well as for the stimulation of bile production [15].

This oil has been reported to contain vitamin A and fatty acids (e.g., palmitic, oleic, myristic, palmitoleic, stearic, linoleic, linolenic acids) [16], which are essential for skin hydration and barrier maintenance, as well as the hydrolipidic mantle [17].

Previously, we demonstrated that supercritical CO_2_ extracts from the leaves of *C. brasiliense* exhibit antimicrobial activity against *Escherichia coli*, *Pseudomonas aeruginosa*, and *Staphylococcus aureus*. They also possess antioxidant activity when compared with a vitamin-E standard [18]. Information about the toxicological potential of *C. brasiliense* is very limited and not sufficient to support its safety. Given the substantial potential of this Brazilian species for wide application in clinical and cosmetic areas, we evaluated the *in vitro* cytotoxicity and phototoxicity of supercritical CO_2_ extract obtained from the leaves of *C. brasiliense*.

## Methods

### Plant material

In January 2011, approximately 25 kg of *C. brasiliense* leaves were collected from Montes Claros (Minas Gerais, Brazil). Leaves were dried in an air-circulating oven at 40°C and then ground in a knife mill. They were stored in plastic bags at room temperature to protect them from humidity. Samples of complete leaves, representative of the species, were identified by the Herbarium of the University of Campinas (São Paulo, Brazil), where a voucher was deposited (reference number UEC 150024).

An apolar extract from *C. brasiliense* was prepared by Chemyunion Química Ltda (São Paulo, Brazil) using a supercritical CO_2_ extraction system comprising a heated extraction column, CO_2_ and co-solvent pumps, a thermostatic bath, and a pressure gauge. These activities were conducted with the approval of the Brazilian Institute of Environment and Renewable Natural Resources, which granted access to genetic resources under number 008/2009 (case number 02001.003785/2011-59).

### Screening of main chemical classes

The phytochemical profile of the crude plant extract was screened using a thin-layer chromatography (TLC) system that tested specific fractions generated, based on differing polarity, during extraction. This procedure fractionated the crude extract into fiber, a neutral extract, moderately polar extract, basic extract, and polar extract according to the method described by Harborne [19].

The chemical profile of the extract was analyzed for the presence of alkaloids, saponins, anthraquinones, steroids, tannins, flavonoids, and phenolic compounds according to conventional colorimetric methods. Compounds from different chemical families were detected by precipitation reactions or staining using reagents specific to each family of compounds.

### Cell culture

Murine fibroblasts (3T3) (ATCC® CCL-92™; American Type Culture Collection, Manassas, VA, USA) were subcultured in Dulbecco’s modified Eagle’s medium (DMEM; Sigma–Aldrich, Saint Louis, MO, USA) supplemented with 0.10% gentamycin, 0.80% Amphotericin B, 0.001% epidermal growth factor, and 10% fetal bovine serum (Sigma–Aldrich). Plates were incubated overnight at 37°C in a 5% CO_2_ incubator. After confluence, cells were trypsinized (0.25% trypsin) for 1 min and seeded on 96-well culture plates (1 × 10^4^ cells/well) for incubation with a *C. brasiliense* supercritical CO_2_ extract (CBSE).

### Cytotoxicity testing using a tetrazolium-based colorimetric assay (XTT)

Cell viability was determined using the XTT assay (Sigma–Aldrich). This test is based on conversion of the yellow tetrazolium hydroxide (sodium 3′-[1-(phenylaminocarbonyl)-3,4-tetrazolium]-bis [4-methoxy-6-nitro] benzene sulfonic acid hydrate) to an orange formazan color by the mitochondrial enzyme succinate dehydrogenase in metabolically active viable cells.

The culture medium was removed from 96-well plates (Nunc, Roskilde, MD, USA) containing cells subcultured previously at 37°C in a 5% CO_2_ incubator. CBSE (0.0001–50.0% *w/v*) well dissolved in culture medium was added to the cells for 48 h at identical conditions of cell culture. After incubation, contents of the wells were aspirated carefully, and the wells were rinsed thrice with Earle’s balanced salt solution (EBSS). XTT (In Cytotox XTT KXT 96.300; Xenometrix AG, Allschwil, Switzerland) was added to the culture, which was incubated at 37°C for 4 h. The absorbance of each well was determined at 450 nm in a microplate reader (Multiscan MS; Labsystems, Helsinki, Finland). The control group comprised untreated wells. Cell death was expressed as a percentage. The half-maximal inhibitory concentration (IC_50_) of CBSE was estimated through analyses of linear interpolation.

### Phototoxicity: the 3 T3 neutral red uptake (NRU) assay

Cells were prepared as described in the cytotoxicity assay using two plates. The 3 T3 NRU test for phototoxicity requires 60-min exposure to sample dilutions in EBSS followed by exposure to UVA light (5 J.cm^-2^; SOL-500 Sun Simulation System; Dr Honle, Planegg, Germany) of one of the plates. The other plate was used as a non-irradiated control. After washing both plates with EBBS, cells were incubated for 24 h at 37°C in a 5% CO_2_ incubator. Plates were washed, incubated with Neutral Red (NR) dye (In Cytotox KRCV 96.300; Xenometrix) and re-washed. NR desorb solution was added and NR absorption measured at 540 nm in a microplate reader (Multiscan MS; Labsystems). Cell viability was calculated for each treatment. Phototoxicity was assessed by comparing the differences in toxicity between negative control plates that had not been exposed to UVA light and test plates exposed to UVA light. Cell death was also expressed as a percentage, and IC_50_ of the CBSE estimated through analyses of linear interpolation.

The Photo-irritation Factor (PIF) was determined according to the following equation:


Equation 1: Calculation of the Photo-irritation Factor.

A second predictor of phototoxicity, the mean photo effect (MPE) was also calculated [20]. The values obtained from PIF and MPE allow categorization as "no photoirritant" (PIF <2 or MPE <0.1), "probable photoirritant" (PIF >2 and <5 or an MPE >0.1 and <0.15) and "photoirritant" (PIF >5 or an MPE >0.15) [21].

### Statistical analyses

Optical density data (in triplicate) are the mean ± standard deviation. A parametric method, one-way analysis of variance (ANOVA), followed by the Dunnett’s test was used to compare data among all groups. A P value <0.05 was considered statistically significant.

## Results and discussion

### Screening of the crude extract

The terpenoid fraction was identified with anisaldehyde reagent, which produced spots of different shades of pink and violet. These spots were also observed with standard H_2_SO_4_, antimony chloride reagent, and 0.2% KMNO_4_.

The phenolic fraction was identified after acid hydrolysis of the dried ground plant extract. Acid hydrolysis was done with 2 M HCl for 30 min. The product workup created an organic fraction that was analyzed by TLC. After separation on silica gel using 45% ethyl acetate in hexane, blue spots were detected with Folin’s reagent.

The main constituents of *C. brasiliense* are flavonoids and terpenoids. These chemical compounds have been identified as being antimicrobial agents [22–25] and contribute to the antioxidant activity demonstrated in our previous study [18].

Flavonoids can interact with the cytoplasmic membrane, inhibiting its function and jeopardizing cellular integrity. They can also inhibit the synthesis of nucleic acids and interrupt bacterial metabolism [26].

Terpenoids are, in general, recognized as being safe. They have been found to inhibit the growth of cancerous cells as well as decrease tumor size, serum cholesterol levels, and microorganism concentrations [27, 28]. Terpenoids have lipophilic characteristics that affect the stability of the cytoplasmic membrane, leading to loss of cellular enzymes and nutrients [29].

Of particular interest, the phytochemical groups, flavonoids and terpenoids, present in CBSE are associated with a range of anti-inflammatory, antimicrobial, anti-edematogenic, and antioxidant activities [30].

### Cytotoxicity and phototoxicity potential

**Figure 1:**
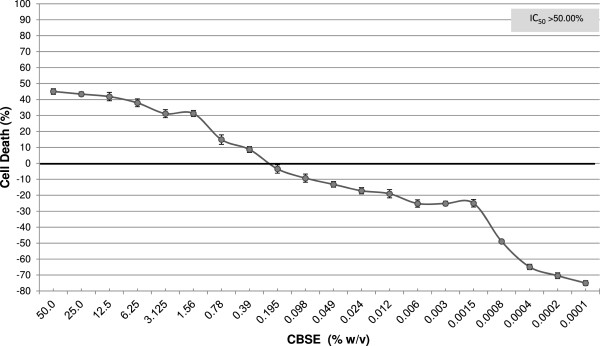
**Evaluation of the cytotoxic potential of CBSE in murine fibroblasts (3 T3) by the XTT assay.** Data are the mean ± SD of three independent experiments. *P <0.05 compared with the control (ANOVA, Dunnett’s test).

The cytotoxic potential of CBSE was evaluated by the XTT assay in murine fibroblasts (3 T3) incubated with the extract (0.0001–50.00%). The extract exhibited negligible cytotoxicity, because its IC_50_ is >50.0% and none of the test concentrations could reduce cell viability to 50% (Figure 1). None of the test concentrations showed a decrease in cell viability compared with the control group (p < 0.05).

**Figure 2:**
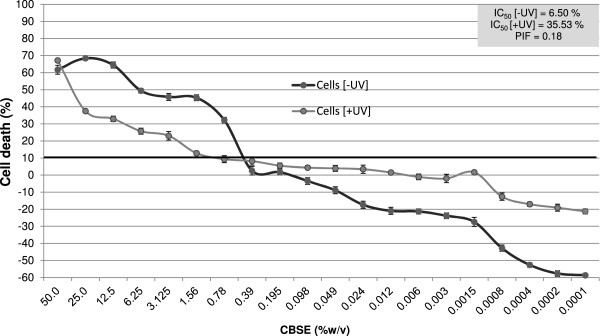
**Evaluation of the phototoxic potential of CBSE in murine fibroblasts (3 T3) by the Neutral Red method (3 T3 RNU).** Data represent the mean ± SD of three independent experiments. *p < 0.05 compared with control (ANOVA, Dunnett’s test).

Figure 2 presents the IC_50_ values for CBSE predicted by linear interpolation in the phototoxicity assay considering dark (6.50% *w/v*) and irradiated (35.53% *w/v*) conditions. According to analyses of the PIF and MPE, CBSE did not exhibit phototoxic potential (PIF =0.18 and MPE = -0.062) in the dose levels tested.

Cytotoxicity was assessed in fibroblast cultures with direct contact between CBSE and basal cells. This is a drastic situation compared with regular use of cosmetics, in which the initial contact occurs in the stratum corneum, a keratinized tissue for skin protection [31].

In general, *in vitro* safety tests are based on disruption of membrane integrity as determined by pigment absorption, to which the cell is (in general) impermeable, or the retention of released pigment by viable cells [32]. Among bioassays, cell toxicity tests are the first to be carried out to predict the toxicity of substances to various tissues [33].

Tests of cell viability provide information about short or immediate responses, thereby enabling determination of the proportion of viable cells after a traumatic procedure. Although *in vitro* tests used to evaluate toxicity cannot completely replace *in vivo* tests, they provide useful information regarding the factors that influence growth and cell survival [34].

Alternatives to animal testing and the identification of validated methods that may decrease the need for animals are currently the subject of intense investigation worldwide. The *in vitro* tests provide an alternative means for the assessment of natural and synthetic ingredients with time efficiency, and cost effectiveness [35].

*C. brasiliense* (Pequi) is used topically for wound dressings [11] and has the potential for skin hydration and barrier maintenance, as well as the hydrolipidic mantle [17]. The relatively few studies focusing on screening of the phototoxic activity of plant extracts employed as cosmetics led us to investigate the effect of CBSE. Studies investigating the cytotoxicity or phototoxicity of CBSE are lacking but Roesler *et al*. [7] showed that the ethanolic extracts of pequi peel and seed were not phototoxic (PIF <5).

*In vitro* cytotoxicity results are used to screen toxicity and to estimate the IC_50_ of chemicals. The NRU phototoxicity assay using 3 T3 cells has been validated by the European Union Reference Laboratory for Alternatives to Animal Testing, and accepted for regulatory use to detect the phototoxic potential of substances. Phototoxic potential is assessed by comparing the differences in toxicity between negative control plates not exposed to UVA and test plates exposed to UVA [7].

As demonstrated by several validation studies, the phototoxic potential of chemicals can be evaluated effectively by *in vitro* methods [36]. However, the neutral red assay (3 T3 NRU) is a validated method for the replacement of tests involving animals [37].

The phototoxicity results obtained by the *in vitro* NRU method are of great importance (especially for cosmetic and pharmaceutical application) because topical formulations are commonly used during the day and involve exposure to the sun and artificial light.

There is a wide search for dermatological actives derived from plants (e.g., fragrance, antimicrobials, antioxidants). However, these plants used in traditional "folk" medicine are associated with cytotoxicity and phototoxicity. In view of this observation, evaluation of the cytotoxic and phototoxic potential of extracts or vegetal actives becomes essential for the development of products for dermatological use [38–42].

We sought to provide information about the biological behavior of CBSE to propose its cosmetic and/or pharmaceutical use. Some researches shown that oils and extracts normally comprising a mixture of components, have better therapeutic activity than the known compounds that represent their main composition. An example of such statement is the antiseptic activity of the essential oil of *Eucalyptus globulus*, higher than the activity shown by its main active constituent isolated, cineol or eucalyptol [43].

In some situations, the results of the tests that evaluate *in vitro* safety might not be related directly to the results obtained through *in vivo* tests. However, a material that induces a reaction in tests involving cell cultures is likely to demonstrate toxic potential if applied to living tissues [44].

Our results are also a screening procedure to assess toxicity. Also, as one of the factors used to estimate the starting dose for *in vivo* acute lethality studies, this approach could reduce the number of animals used in *in vivo* studies and minimize the number of animals that receive lethal doses.

The safety of *Caryocar brasiliense* supercritical extract has not been well reported at scientific literature and the lack of reliable data does not allows a good comparison of our results. Thus, additional researches are needed to explore other toxicity pathways of this species that has a great potential of use in cosmetic and/or pharmaceutical products.

## Conclusions

Under the test conditions described, *C. brasiliense* (pequi) extract obtained by supercritical CO_2_ extraction did not appear to have cytotoxic and phototoxic hazards. This finding suggests that the extract may be useful for the development of cosmetic and/or pharmaceutical products. However, more detailed research is needed to explore other toxicity pathways.
